# Nomogram for prediction of hearing rehabilitation outcome in children with congenital sensorineural hearing loss after cochlear implantation

**DOI:** 10.1016/j.heliyon.2024.e29529

**Published:** 2024-04-16

**Authors:** Xi Deng, Xueqing Yang, Meiru Bu, Anzhou Tang, Huiting Zhang, Liling Long, Zisan Zeng, Yifeng Wang, Ping Chen, Muliang Jiang, Bihong T. Chen

**Affiliations:** a Department of Radiology, The First Affiliated Hospital of Guangxi Medical University, No.6 Shuangyong Road, Nanning, 530021, Guangxi, PR China; b Department of Otorhinolaryngology Head and Neck Surgery, The First Affiliated Hospital of Guangxi Medical University, No.6 Shuangyong Road, Nanning, 530021, Guangxi, PR China; c MR Research Collaboration, Siemens Healthineers Ltd., 430000, Wuhan, PR China; d Institute of Brain and Psychological Sciences, Sichuan Normal University, No. 5, Jing'an Road, Chengdu, 610066, Sichuan, PR China; e Department of Diagnostic Radiology, City of Hope National Medical Center, 1500 E, Duarte, CA, 91010, USA

**Keywords:** Brain magnetic resonance imaging (MRI), Cochlear implantation, Congenital sensorineural hearing loss, Nomogram, Power spectrum analysis, Rehabilitation outcome, Resting-state functional MRI

## Abstract

**Background:**

Reliable predictors for rehabilitation outcomes in patients with congenital sensorineural hearing loss (CSNHL) after cochlear implantation (CI) are lacking. The purchase of this study was to develop a nomogram based on clinical characteristics and neuroimaging features to predict the outcome in children with CSNHL after CI.

**Methods:**

Children with CSNHL prior to CI surgery and children with normal hearing were enrolled into the study. Clinical data, high resolution computed tomography (HRCT) for ototemporal bone, conventional brain MRI for structural analysis and brain resting-state fMRI (rs-fMRI) for the power spectrum assessment were assessed. A nomogram combining both clinical and imaging data was constructed using multivariate logistic regression analysis. Model performance was evaluated and validated using bootstrap resampling.

**Results:**

The final cohort consisted of 72 children with CSNHL (41 children with poor outcome and 31 children with good outcome) and 32 healthy controls. The white matter lesion from structural assessment and six power spectrum parameters from rs-fMRI, including Power4, Power13, Power14, Power19, Power23 and Power25 were used to build the nomogram. The area under the receiver operating characteristic (ROC) curve of the nomogram obtained using the bootstrapping method was 0.812 (95 % CI = 0.772–0.836). The calibration curve showed no statistical difference between the predicted value and the actual value, indicating a robust performance of the nomogram. The clinical decision analysis curve showed a high clinical value of this model.

**Conclusions:**

The nomogram constructed with clinical data, and neuroimaging features encompassing ototemporal bone measurements, white matter lesion values from structural brain MRI and power spectrum data from rs-fMRI showed a robust performance in predicting outcome of hearing rehabilitation in children with CSNHL after CI.

## 1 Introduction

Congenital sensorineural hearing loss (CSNHL) is the most common sensory deficit due to a defect in the sound-to-nerve conduction system [1]. According to an epidemiological survey, about 1.2–1.7 cases of permanent CSNHL occur per 1000 births, and 30 % of these patients have severe hearing loss [2]. Children with CSNHL will likely experience significant delays in speech and language development, resulting in poor language skills by preschool age, with negative social, academic, and vocational consequences if no timely treatment [3,4]. Cochlear implantation (CI) is an optimal treatment for individuals with severe hearing loss, and studies have shown that it improves speech and language performance compared to other treatments within the first 4 years of life [2,5]. However, the outcome of hearing and speech rehabilitation after CI is variable and some children with CSNHL do not recover as expected [[6], [7], [8]]. It is prudent to develop non-invasive markers to assess the outcome of hearing and speech rehabilitation after CI.

Literature has reported that clinical information such as premature birth [9,10] and brain structural MRI assessment such as white matter lesions [11,12] being related to hearing loss in newborns. Additional factors such as age at CI surgery [[13], [14], [15]], preoperative speech rehabilitation training [16], the width of bony cochlear nerve canal [17,18] and the diameter of internal auditory canal [19] have been shown to be related to the rehabilitation outcome after CI surgery. One prior study of children with CSNHL used brain functional connectivity from resting-state functional MRI (rs-fMRI) to develop a machine learning model for prediction of prognosis following CI [20]. Our own published study using rs-fMRI reported alterations of regional homogeneity in auditory, visual, motor and other related brain cortex in patients with CSNHL as compared to the healthy controls [21]. However, there are no known validated models to predict specific brain regions being associated with hearing rehabilitation. Therefore, more work is needed to improve the assessment of patients after CI.

Nomogram relies on a set of variables to make individualized predictions rather than generalizing to population risk groups, which has gained recognition for its potential usefulness to predict patient survival, evaluate treatment effects, identify risk factors and guide personalized treatment decisions [22,23]. Previous studies have used nomogram to study the prognosis of sudden deafness of unknown cause in adults, and the results show that nomograms could effectively assist in predicting prognosis of regaining hearing and optimizing treatment options based on the protective or risk factors identified by the nomogram [24,25]. Low-frequency oscillation (LFO) is a neurophysiologic index related to spontaneous neural activity [26,27] and the amplitude of LFO can be assessed on rs-fMRI with power spectrum [28], which is one of most significant parameter of LFO [26,27,29]. Because of its greater robustness, power spectrum has been used to study brain network changes during development [30], sensorimotor network alterations in patients with myotonic dystrophy type 1 [31] and discrimination between good versus poor recovery after subacute stroke [32]. However, there is limited literature on power spectrum analysis and nomogram prediction in patients with CSNHL.

Here, we performed a prospective neuroimaging study on rehabilitation outcome of children with CSNHL after CI surgery, and compared them to children with normal hearing. In addition, we also compared the subgroups of children with CSNHL in terms of their outcome after CI. We assessed their clinical data, high resolution computed tomography (HRCT) for ototemporal bone, conventional brain MRI for structural analysis and rs-fMRI parameters for power spectrum analysis prior to CI, aiming to develop a model to predict the hearing rehabilitation outcome of children with CSNHL after CI. Our approach should assist in clinical decision making for the most optimal personalized treatment to achieve hearing rehabilitation after CI in children with CSNHL.

## 2 Methods

### 2.1 Participants

Children with CSNHL aged 0–13 years prior to CI surgery were recruited into this study from January 2015 to October 2019, and children with normal hearing from the community were enrolled as healthy controls. The eligibility criteria for the patient group were the following: children between 0 and 13 years of age with profound CSNHL, a mean auditory brainstem response (ABR) threshold of >91 dB (dB), auditory steady-state evoked potential >80 dB, and hearing loss within the range of 250–4000 Hz. The exclusion criteria were the following: severe neurological disorders such as epilepsy, severe cognitive impairment such as autism or hyperkinetic syndrome, history of contraindications for MRI such as having a cardiac pacer and orbital metal, and poor-quality MRI scans that were rendered suboptimal for data analysis. A portion of this cohort was included in our prior study of regional homogeneity but no predictive modeling has been published from this cohort yet [21].

**Fig. 1:**
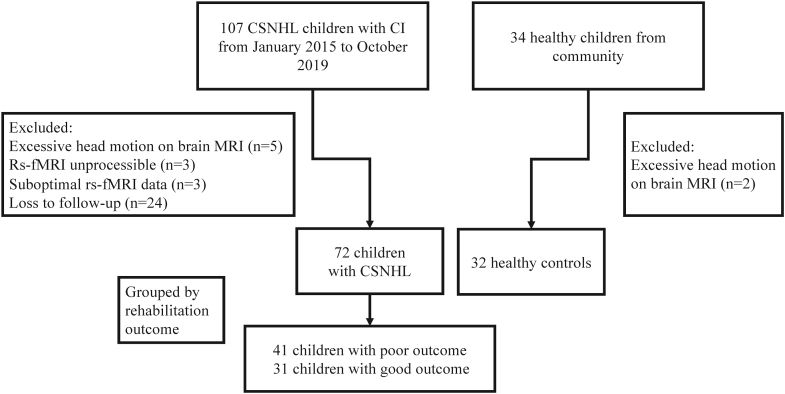
Study participant enrollment process. Abbreviations: CSNHL = congenital sensorineural hearing loss, CI = cochlear implantation, MRI = magnetic resonance imaging, rs-fMRI = resting-state functional MRI.

The category of auditory perception (CAP) is an effective tool for evaluating hearing and speech rehabilitation. We divided the enrolled children with CSNHL into two groups with good and poor rehabilitation outcomes according to the score one year after CI surgery [33]. Fig. 1 presents the study participant enrollment process. The CAP scores of children with CSNHL one-year after CI surgery were assessed by an experienced otolaryngologist and two speech rehabilitation specialists, and the three experts reconciled the differences in their assessment through discussion among themselves in case of conflicting results. If CSNHL children achieved a CAP score of 5 points or more one-year post-CI, they were considered as having a good rehabilitation outcome and would be in the good outcome group. Otherwise, they would be in the poor outcome group. The specific grouping information of the rehabilitation effect is available in Table S1. This study was approved by our hospital ethics committee and informed consents were obtained from all participants’ parents or legal guardians.

### 2.2 Neuroimaging data acquisition

All brain MRI images were acquired on the same Siemens Magnetom Verio 3T MRI scanner (Siemens Healthcare; Erlangen, Germany) using a 12-channel phased-array head coil (Siemens). The head position was stabilized with sponge support. All participants were sedated for the MRI scan with oral chloral hydrate under the care of medical professionals. There were no complications from the oral sedatives.

The rs-fMRI data was obtained with a gradient-echo planar imaging sequence with the following parameters: repetition time/echo time = 2000/30 ms, 36 slices, 64 × 64 matrix, 90° flip angle, 24 cm field of view, 4 mm slice thickness, 0.4 mm gap, and 250 vol (500 s). In addition, conventional structural brain MRI data including a T2-weighted fluid-attenuated inversion recovery (FLAIR) sequence and a T1-weighted three-dimensional magnetization-prepared rapid gradient echo (MP-RAGE) sequence were acquired. A T2-FLAIR routine MRI scans were obtained with the following protocol: slice thickness, 5.0 mm; slice interval, 0 mm; repetition time, 8000 ms; echo time, 103 ms; slices, 24; 21 cm field of view; 150° flip angle. A three-dimensional magnetization-prepared rapid gradient echo (MP-RAGE) sequence was obtained with the following protocol: slice thickness, 0.9 mm; slice interval, 0 mm; repetition time, 1700 ms; echo time, 2.9 ms; inversion time, 900 ms; matrix, 256 × 256; field of view, 220 × 220 mm^2^; and slice number, 140. The FLAIR data was used for assessing white matter lesions and incidental brain pathology. The MP-RAGE data was used for registration of rs-fMRI images to Montreal Neurological Institute (MNI) standard space.

### 2.3 Conventional MRI imaging assessment

Two senior neuroradiologists, with 15 and 25 years of experience, blinded to clinical information independently assessed all participants for white matter lesions on the conventional structural brain MRI. In addition, they also measured the width of bony cochlear nerve canal (BCNC) and the diameter of internal auditory canal (IAC) on high resolution computed tomography (HRCT) for ototemporal bone. Specifically, the width of the BCNC was measured at the fundus of the IAC by drawing two straight lines along the fundus and the base of the modiolus of cochlea, and the midline distance was deemed as the width of BCNC according to a prior study [19]. The diameter of the midportion of the IAC in the axial plane was measured at the level of porous acoustics in HRCT images. The measurement was taken along a line orthogonal to the long axis of the IAC from the posterior margin to the anterior wall. We evaluated the agreement between the two neuroradiologists using intraclass correlation coefficients (ICCs). The ICCs between the results of two neuroradiologists were greater than 0.8, which was considered adequate.

### 2.4 rs-fMRI data analysis

The time series of rs-fMRI data were filtered into four frequency bands with the decoding rhythms of the brain system (DREAM) software [34]: slow-5 (0.012–0.030 Hz), slow-4 (0.030–0.082 Hz), slow-3 (0.082–0.224 Hz), and slow-2 (0.224–0.25 Hz). Then, rs-fMRI data were preprocessed with Data Processing & Analysis for Brain Imaging package (DPABI, V6.1, http://rfmri.org/dpabi) based on the MATLAB 2021b platform [35]. Briefly, the first 10 vol were discarded to ensure signal stabilization, subject adaptation, slice timing, and spatial realignment. Participants with excessive head motion with mean frame-wise displacement (FD) [36] larger than 0.2 were excluded [37].

All images were normalized to the standard space on the MNI template and were resampled to 3 × 3 × 3 mm^3^, which had been considered appropriate for brain normalization for young children through adolescence [38,39]. Subsequently, linear trend, white matter signal, cerebrospinal fluid signal, and Friston 24 motion parameters were regressed to reduce effects of head movement and non-neuronal information [40]. Subsequently, the normalized images were spatially smoothed with a 6-mm full width at half maximum (FWHM) Gaussian kernel.

After preprocessing using the DPABI package, the time series of each brain region defined by Brainnetome atlas [41] containing 246 sub regions of the bilateral hemispheres was converted to the frequency domain by using a Fast Fourier Transform (FFT), and the power spectrum of each brain region was obtained with a frequency resolution equal to 0.0021 Hz (0.0021Hz–0.25Hz). Eventually, power spectral values for each subject were obtained for each frequency point and for the brain region.

Prior to statistical analysis, age and gender were regressed from power spectrum values. Then, the residuals of power spectrum values for each frequency point in the brain area were subjected to independent sample *t*-test in the patient group and the control group, respectively, to obtain frequency points and brain areas with significant differences between these two groups. The results were corrected by false discovery rate (FDR) method [42] to reduce the probability of type 1 error. In addition, this process was also performed on the two sub groups which were defined by good or poor rehabilitation outcome. The frequency points and brain areas with significant differences in power spectrum values between the two subgroups were obtained.

### 2.5 Predictive modeling

**Fig. 2:**
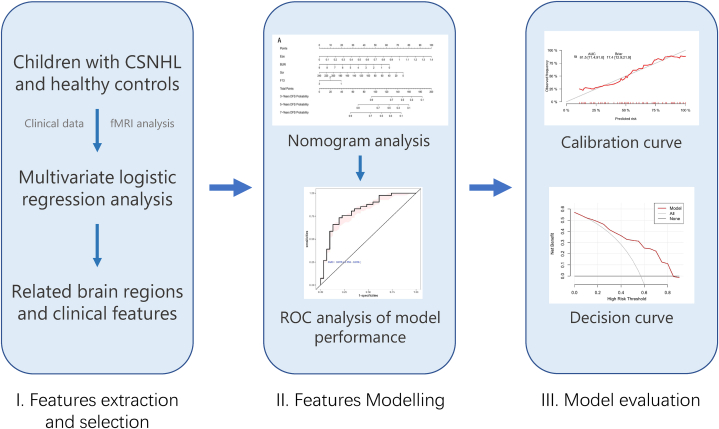
Workflow diagram for building the prediction model. Multivariate logistic regression analysis was performed to identify the relevant brain regions and clinical features. The nomogram, receiver operating characteristic (ROC) curve, calibration curve, and decision curve assessed the model performance.

Univariate logistic regression was performed to analyze the risk factors associated with hearing rehabilitation outcome. The model was constructed by the following variables, including gender, current age, age at CI, inner ear malformation, otitis media, pneumonia, preoperative speech rehabilitation training, preoperative hearing aid use, premature birth, internal auditory canal diameter (the average of left and right ears), bony cochlear nerve canal diameter (the average of left and right ears) and the power spectrum values of the brain regions with significant different frequency bands. The model construction was completed in R software (version 4.0.3, R Foundation for Statistical Computing, Vienna, Austria). The final model selection was conducted using a backward stepdown selection with the Akaike information criterion (AIC). The nomogram, decision curve analysis and calibration curves were plotted using the rms package in R. Receiving operation curves (ROC) were plotted by the pROC package in R. The subsequent multivariate logistic regression model was used to calculate the risk score and for building the final nomogram prognostic model. All internal validations were performed using bootstrapping method with 1000 resamples. Fig. 2 shows the workflow diagram for predictive modeling.

### 2.6 Statistical analysis

Statistical analysis for clinical and demographic data was performed with the SPSS software (version 26.0, SPSS Inc., Chicago, IL, USA). Two-sample *t*-test was used for continuous variable, while the Fisher's exact test was used for categorical variables. A two-sided p-value less than 0.05 was considered as statistically significant.

## 3 Results

### 3.1 Demographic and clinical information

A total of 107 children with CSNHL aged 0–13 years prior to CI surgery were prospectively enrolled into this study, and 34 children with normal hearing were enrolled as healthy controls. In the group with CSNHL, 35 participants were excluded for the following reasons: excessive head motion with mean FD larger than 0.2 (n = 5), rs-fMRI data not processible (n = 3), suboptimal rs-fMRI data (n = 3) and loss to follow-up (n = 24). In addition, two healthy controls were excluded due to excessive head motion with mean FD larger than 0.2 (n = 2). The final cohort consisted of 72 children with CSNHL (31 children with good outcome and 41 children with poor outcome) and 32 healthy controls (Fig. 1). Table 1 presents the demographic and clinical information of the study cohort.

**Table 1:** Demographic and clinical data of patients with congenital sensorineural hearing loss (CSNHL) having poor and good rehabilitation outcome after cochlear implant (CI).

Characteristics	Good (n = 31) Mean ± SD	Poor (n = 41) Mean ± SD	P-value
Age	3.10 ± 2.14	3.50 ± 2.29	0.436
Age at CI surgery	3.54 ± 2.15	3.99 ± 2.27	0.405
Bony cochlear nerve canal (BCNC) measurement	2.90 ± 0.40	2.69 ± 0.58	0.100
Internal auditory canal (IAC) measurement	4.96 ± 0.78	5.29 ± 1.05	0.141
Gender			0.634
Male	14	22	
Female	17	19	
Premature birth			1
Yes	1	1	
No	30	40	
White matter lesion			0.043
Yes	6	18	
No	25	23	
Inner ear malformation			1.000
Yes	9	13	
No	22	28	
Otitis media			1.000
Yes	4	5	
No	27	36	
Pneumonia at birth			0.227
Yes	1	5	
No	30	36	
Preoperative hearing aid use			0.751
Yes			
No			
Preoperative speech rehabilitation training			0.813
Yes	18	25	
No	13	16	

### 3.2 Power spectrum data based on outcome

Analysis of power spectrum values from the rs-fMRI data identified 27 brain regions and frequency points with significant differences between the good and poor outcome subgroups in the children with CSNHL (Table 2). In Table 2, the 27 brain regions and frequency points were sequentially numbered as Power 1 to Power 27. The frequencies of these differences were ranged from 0.013 Hz to 0.231 Hz, and the corresponding power spectrum values were included in the construction of the predictive model.

**Table 2:** Brain regions and frequencies from power spectrum analysis of resting-state functional MRI showing significant differences, and univariate and multivariate analysis in power spectrum power of rehabilitation outcome between the good and the poor outcome subgroups of children with congenital sensorineural hearing loss (CSNHL) after cochlear implant (CI).

Subject	Frequency (Hz)	Brainnetome atlas area	Good (n = 31) Mean ± SD	Poor (n = 41) Mean ± SD	P-value	Univariate analysis						Multivariate analysis					
						B	SE	OR	95 % CI	Z	P	B	SE	OR	95 % CI	Z	P
Power1	0.152	3	4.74 ± 2.84	7.59 ± 7.33	0.045	0.141	0.074	1.15	1–1.33	1.894	0.058						
Power2	0.054	4	7.2 ± 4.16	9.55 ± 4.93	0.036	0.124	0.061	1.13	1–1.28	2.013	0.044						
Power3	0.033	5	9.44 ± 4.93	13.09 ± 9.04	0.046	0.084	0.043	1.09	1–1.18	1.933	0.053						
Power4	0.063	11	7.32 ± 3.21	10.38 ± 5.67	0.009	0.152	0.062	1.16	1.03–1.31	2.445	0.014	0.178	0.072	1.19	1.04–1.38	2.453	0.014
Power5	0.063	13	7.22 ± 3.82	10.01 ± 6.09	0.028	0.117	0.056	1.12	1.01–1.25	2.09	0.037						
Power6	0.058	14	6.86 ± 3.35	10.14 ± 8.45	0.045	0.111	0.058	1.12	1–1.25	1.908	0.056						
Power7	0.058	15	4.84 ± 2.33	6.55 ± 3.58	0.023	0.201	0.094	1.22	1.02–1.47	2.139	0.032						
Power8	0.058	16	4.71 ± 1.9	6.90 ± 3.43	0.002	0.322	0.118	1.38	1.1–1.74	2.725	0.006						
Power9	0.060	16	4.75 ± 1.79	6.15 ± 2.91	0.021	0.28	0.128	1.32	1.03–1.7	2.194	0.028						
Power10	0.088	16	3.85 ± 1.57	5.08 ± 2.95	0.039	0.241	0.124	1.27	1–1.62	1.944	0.052						
Power11	0.102	16	3.98 ± 1.05	5.01 ± 2.61	0.043	0.272	0.141	1.31	1–1.73	1.938	0.053						
Power12	0.106	16	3.74 ± 1.26	4.95 ± 3.16	0.047	0.256	0.138	1.29	0.99–1.69	1.859	0.063						
Power13	0.154	16	3.54 ± 1.07	4.20 ± 1.60	0.049	0.364	0.19	1.44	0.99–2.09	1.914	0.056	−0.572	0.405	0.56	0.26–1.25	−1.413	0.158
Power14	0.231	16	2.73 ± 0.65	3.39 ± 1.44	0.02	0.793	0.359	2.21	1.09–4.47	2.212	0.027	0.717	0.417	2.05	0.9–4.64	1.722	0.085
Power15	0.094	19	4.02 ± 1.73	5.38 ± 2.87	0.022	0.278	0.13	1.32	1.02–1.7	2.141	0.032						
Power16	0.173	20	3.17 ± 1.30	4.10 ± 1.75	0.016	0.437	0.195	1.55	1.06–2.27	2.243	0.025						
Power17	0.048	23	7.85 ± 4.40	11.52 ± 7.54	0.018	0.114	0.05	1.12	1.02–1.24	2.264	0.024						
Power18	0.063	23	6.10 ± 2.78	8.37 ± 5.37	0.035	0.136	0.068	1.15	1–1.31	2.015	0.044						
Power19	0.154	23	4.24 ± 2.38	5.90 ± 3.64	0.031	0.205	0.103	1.23	1–1.5	1.986	0.047	0.229	0.156	1.26	0.93–1.71	1.467	0.142
Power20	0.213	23	3.36 ± 1.17	4.36 ± 2.46	0.041	0.371	0.203	1.45	0.97–2.16	1.824	0.068						
Power21	0.215	23	3.23 ± 1.21	4.55 ± 3.37	0.041	0.379	0.21	1.46	0.97–2.21	1.806	0.071						
Power22	0.063	31	5.77 ± 2.06	7.85 ± 4.47	0.019	0.194	0.09	1.21	1.02–1.45	2.16	0.031						
Power23	0.225	31	3.48 ± 1.57	4.44 ± 1.96	0.030	0.329	0.159	1.39	1.02–1.9	2.06	0.039	0.444	0.211	1.56	1.03–2.36	2.097	0.036
Power24	0.208	107	3.90 ± 1.26	5.01 ± 2.39	0.022	0.333	0.154	1.4	1.03–1.89	2.163	0.031						
Power25	0.013	123	13.42 ± 7.88	17.83 ± 8.84	0.031	0.065	0.031	1.07	1–1.13	2.087	0.037	0.087	0.037	1.09	1.01–1.17	2.343	0.019
Power26	0.013	124	18.04 ± 14.20	27.06 ± 17.22	0.021	0.04	0.018	1.04	1.01–1.08	2.19	0.028						
Power27	0.015	129	9.69 ± 4.61	13.39 ± 9.39	0.048	0.077	0.042	1.08	0.99–1.17	1.843	0.065						

Notes. — B = regression coefficient, OR = odds ratio, SE = standard error values, CI = confidence interval, Z = z scores, P = p value.

### 3.3 Univariate and multivariate logistic regression data

**Fig. 3:**
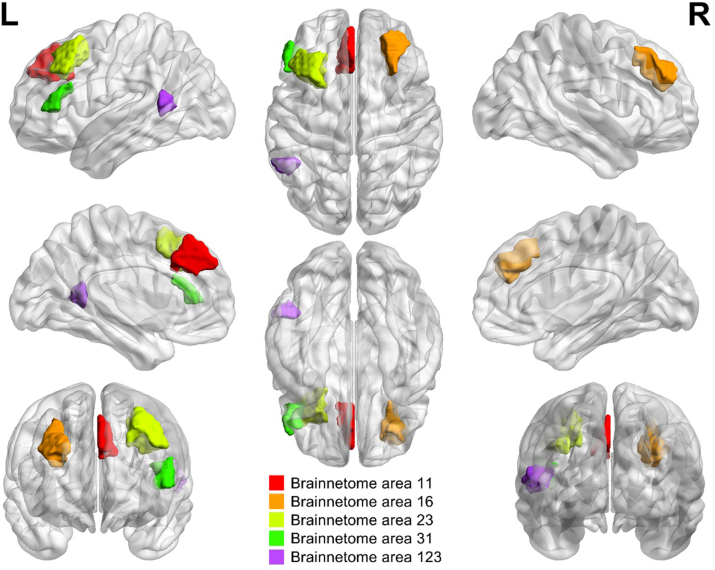
The highlighted areas in the brain from power spectrum analysis of resting-state functional MRI present the brain regions with significant differences between the good and poor outcome subgroups of children with congenital sensorineural hearing loss (CSNHL) after cochlear implant (CI). L, Left; R, Right.

Univariate and multivariate logistic regression analyses were performed to select the effective prognostic characteristics. The univariate analysis revealed that the power spectrum values of Power2, 4, 5, 7, 8, 9, 14, 15, 16, 17, 18, 19, 24, 25, 26, and white matter lesions were significant risk factors for outcome prediction (Table 3). AIC was used in multivariate analysis to identify the effective independent risk factors. Multivariate logistic regression and backward stepdown selection were used to analyze these factors. The following variables remain statistically significant and were considered as independent risk factors for poor rehabilitation outcome for children with CSNHL after CI: Power4 (f = 0.063Hz, Left superior frontal gyrus, A9m, medial area 9), Power13 (f = 0.154Hz, Right middle frontal gyrus, A9/46d, dorsal area 9/46), Power14 (f = 0.231Hz, Right middle frontal gyrus, A9/46d, dorsal area 9/46), Power19 (f = 0.154 Hz, Left middle frontal gyrus, A8vl, ventrolateral area 8), Power23 (f = 0.225 Hz, Left inferior frontal gyrus, inferior frontal sulcus), Power25 (f = 0.013Hz, Left posterior superior temporal sulcus, caudoposterior superior temporal sulcus) and a conventional MRI feature such as WMLs (Yes or no). The brain regions for the relevant power spectrum values were visualized in Fig. 3.

**Table 3:** Univariate and multivariate analysis in clinical characteristic of rehabilitation outcome of children with CSNHL after cochlear implant (CI) surgery.

Variables	Univariate analysis						Multivariate analysis					
	B	SE	OR	95 % CI	Z	P	B	SE	OR	95 % CI	Z	P
Age	0.088	0.115	1.09	0.87–1.37	0.764	0.445						
Age at CI surgery	0.097	0.116	1.1	0.88–1.38	0.837	0.403						
Bony cochlear nerve canal (BCNC) measurement	−0.938	0.586	0.39	0.12–1.23	−1.599	0.11						
Internal auditory canal (IAC) measurement	0.394	0.27	1.48	0.87–2.52	1.461	0.144						
Premature birth	−0.288	1.435	0.75	0.05–12.49	−0.201	0.841						
Gender	−0.341	0.478	0.71	0.28–1.82	−0.713	0.476						
Preoperative speech rehabilitation training	0.121	0.485	1.13	0.44–2.92	0.249	0.803						
White matter lesions	1.182	0.553	3.26	1.1–9.64	2.138	0.033	1.499	0.681	4.48	1.18–17	2.199	0.028
Preoperative hearing aid use	0.336	0.634	1.4	0.4–4.85	0.531	0.596						
Inner ear deformity	0.127	0.519	1.13	0.41–3.14	0.244	0.807						
Otitis media	−0.065	0.718	0.94	0.23–3.83	−0.09	0.928						
Pneumonia at birth	1.427	1.123	4.17	0.46–37.64	1.271	0.204						

Notes. — B = regression coefficient, OR = odds ratio, SE = standard error values, CI = confidence interval, Z = z scores, P = p value.

### 3.4 Predictive modeling and nomogram

The seven independent risk factors from multivariate analysis including Power4, 13, 14, 19, 23, 25 and white matter lesions were used to construct a predictive model and nomogram. The rehabilitation outcome predictive model after CI was constructed as the equation:

Logit(p)=−5.920+1.499×(Whitematterlesionsequals0or1)+0.178×Power4+(−0.572)×Power13+0.717×Power14+0.229×Power19+0.444×Power23+0.887×Power25

**Fig. 4:**
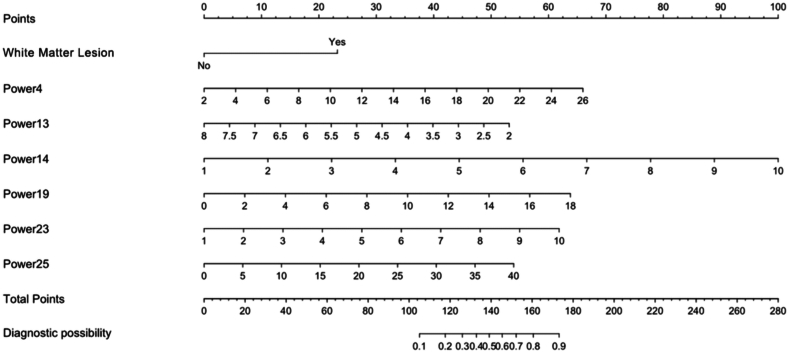
Nomogram for probability of a poor hearing rehabilitation outcome. The nomogram scores are assigned to the seven factors as above including White Matter Lesion, Power4, Power13, Power14, Power19, Power23 and Power25. The higher the total score, the higher the probability of a poor hearing rehabilitation. Note: The brain regions these power spectra represent are as follows: Power 4 for left superior frontal gyrus, Power13 for right middle frontal gyrus, Power14 for right middle frontal gyrus, Power19 for left middle frontal gyrus, Power23 for left inferior frontal gyrus & inferior frontal sulcus, and Power25 for left posterior superior temporal sulcus & caudoposterior superior temporal sulcus. Power4 and Power25 reflect gray matter-related neural activity, while Power13, Power14, Power 19 and Power23 reflect white matter-related neural activity.

The nomogram was created based on the regression coefficients listed above. Correspondingly, the scores of the nomogram were assigned to the seven factors. The higher the total score, the higher the probability of poor hearing rehabilitation. The nomogram for probability of poor hearing rehabilitation was presented in Fig. 4.

### 3.5 Validation and evaluation of nomogram

**Fig. 5:**
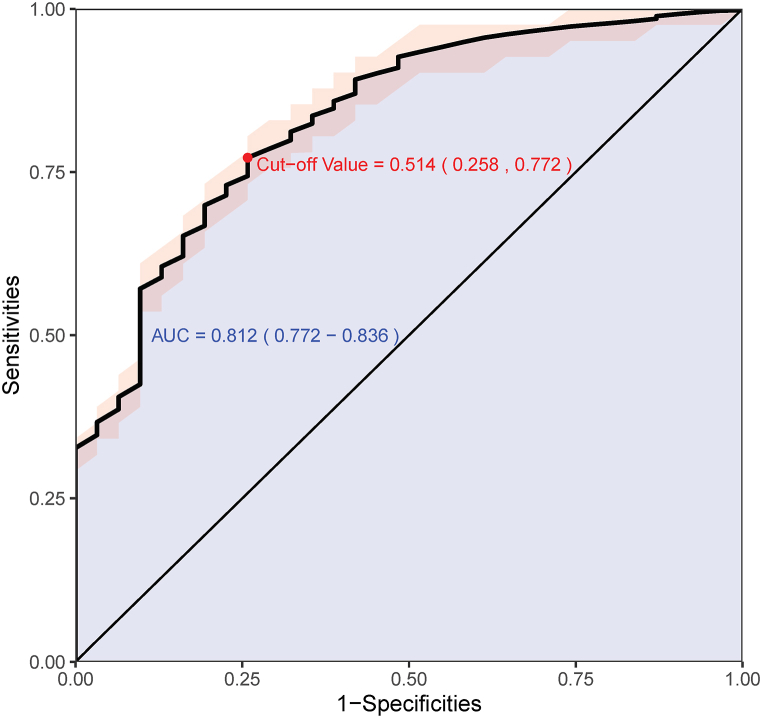
Receiver operating characteristic (ROC) curve and area under the curve (AUC) of the prediction model (Blue shaded area, AUC = 0.812 [95 % CI = 0.772–0.836]) validated by bootstrapping method with 1000 resamples, and the cut-off value of the ROC curve being 0.514. Red shaded area in the plot indicates the confident interval of 1000 times of internal validation. (For interpretation of the references to colour in this figure legend, the reader is referred to the Web version of this article.)

**Fig. 6:**
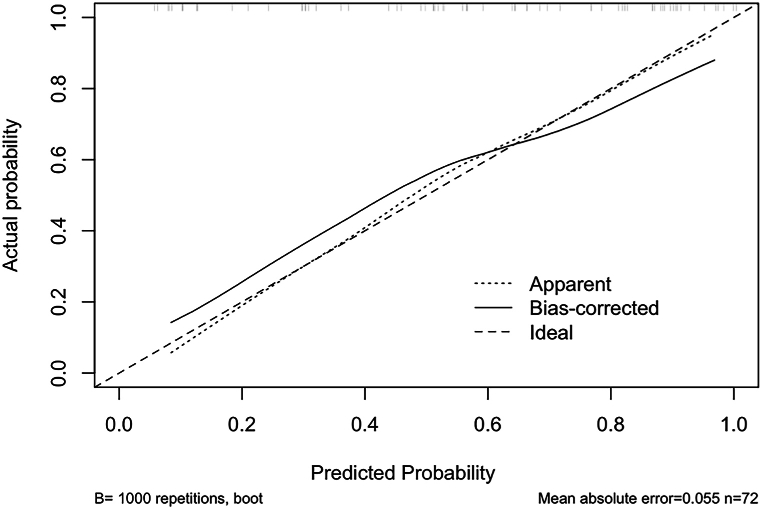
Calibration curve of the predictive model showing the model being well calibrated. The small bias of the predicted results from the actual results indicated a good predictive ability of the model.

**Fig. 7:**
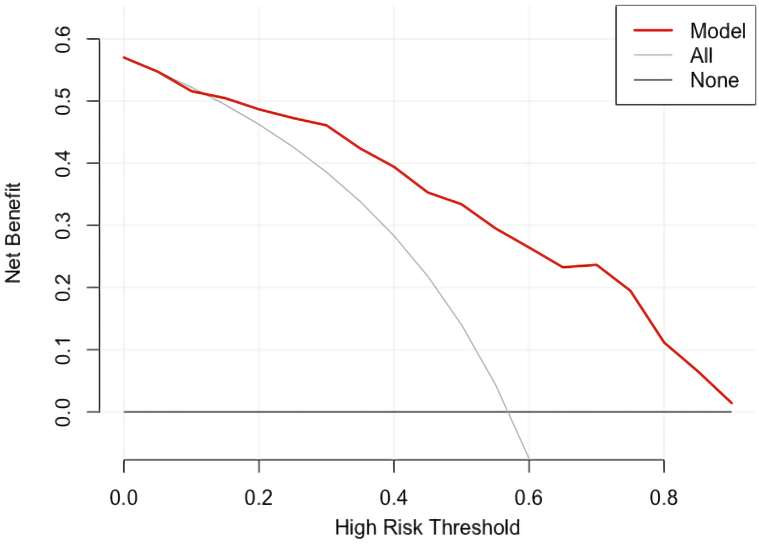
Decision curve of the predictive model (red curve) being higher than the clinical non-intervention curve (gray curve) indicating higher clinical value of intervention in children with congenital sensorineural hearing loss (CSNHL) after cochlear implant (CI) and with more benefit than in the case of non-intervention. (For interpretation of the references to colour in this figure legend, the reader is referred to the Web version of this article.)

Internal validation of combined models was performed using bootstrapping method with 1000 resamples. The area under the ROC curve (receiver operating characteristic curve) was 0.812 (95 % CI = 0.772–0.836) (Fig. 5). Calibration test and clinical decision analysis were performed on this nomogram, also using the bootstrapping method (Fig. 6, Fig. 7). The calibration curve showed no statistical difference between the value of the prediction by the model and the actual value, indicating the prediction model having high degree of calibration. The clinical decision analysis curve showed that the benefits were more significant than side effects after most patients received treatment according to this model, indicating the model having a high clinical value.

## 4 Discussion

In this study, we developed a prediction model combining clinical data, HRCT for ototemporal, conventional brain MRI features, and rs-fMRI parameters to predict the outcome of hearing rehabilitation in children with CSNHL after CI surgery. The combined model demonstrated robust performance and showed the potential to be used as a non-invasive tool for outcome prediction after CI.

Published literature has reported using rs-fMRI parameter such as functional connectivity to build a model for identification of sensorineural hearing loss and for prognostic prediction after cochlear implantation [20]. However, there is no existing model that incorporates clinical data and imaging features. In addition, the published models did not map the specific brain regions associated with rehabilitation outcome [20]. Moreover, functional connectivity may overestimate the genuine effect in terms of neuronal interaction [43], which has motivated further evaluation of rs-fMRI parameters in addition to connectivity. We assessed for independent outcome prognostic factors in a logistic regression model based on analysis of clinical data, HRCT for ototemporal bone, conventional brain MRI features, and power spectrum values from rs-fMRI. Our analysis had merit in its comprehensive approach to encompassing all available data for the most robust performance of a prediction model.

We identified a brain structural MRI feature, i.e., WML, being a risk factor with the highest OR value, indicating its important position in the model and its significance for prognosis of patients with CSNHL after CI. Our study results were consistent with prior studies. For instance, one study reported an association between the severity of WML and the hearing recovery in patients with sudden sensorineural hearing loss, i.e., the higher the Fazekas score for WML, the less the probability of hearing recovery [44]. Another study showed that the presence of WML could be linked to a poor hearing recovery rate [45].

Our study showed the power spectrum value for the inferior frontal gyrus being increased in preoperative rs-fMRI of patients with CSNHL who had poor hearing rehabilitation outcome after CI. Although the children used hearing aids prior to CI, the use of hearing aids did not qualify as an independent risk factor in the multivariate regression analysis. Therefore, the use of hearing aids did not seem to have a significant effect on the postoperative rehabilitation outcomes of CI. This may be attributed to the fact that the amplification function of hearing aids cannot improve sound conduction disorders and subsequently cannot influence or facilitate the reconstruction of responsible brain regions. Furthermore, an auditory stimulation fMRI study showed that children with poor rehabilitation outcome after CI surgery had functional specialization of auditory cortical areas for visual processing [46], resulting in diminished auditory cortical function and thus poor outcome. Therefore, we assume that the poor hearing rehabilitation outcome due to auditory deprivation and functional specialization of auditory cortical areas during the early sensitive period in our study of young children with poor outcome, resulting in the compensatory activation of hearing-related brain regions with increased intrinsic brain activity and power spectrum values on rs-fMRI. It may also be partly due to the brain response to the reorganization of the auditory-language network in children with CSNHL [47].

Different sub-frequency bands reflect different spontaneous neural activity of the brain [[48], [49], [50]]. There were four frequency bands identified in our study, i.e., the slow-5 (0.012–0.030 Hz), slow-4 (0.030–0.082 Hz), slow-3 (0.082–0.224 Hz), and slow-2 (0.224–0.25 Hz). The slow-4 and slow-5 frequency bands mainly reflected the gray matter-related LFO amplitudes [50], whereas the slow-2 and slow-3 frequency bands mainly reflected the white matter-related LFO amplitudes and physiological noises [49,50]. In our power spectrum value analysis, the Power13, Power14, Power 19 and Power23 derived from rs-fMRI corresponded to brain activity changes in bilateral middle frontal gyrus and left inferior frontal gyrus, respectively, which were located in the slow-2 or slow-3 frequency bands reflecting white matter-related neural activity. Our previous study based on tract-based spatial statistics showed significant alterations in the frontal white matter microstructure in children with CSNHL, which may have affected frontal lobe function and thus hearing recovery [51]. Power4 and Power25 corresponded to the left superior frontal gyrus and left posterior superior temporal sulcus respectively, which were located in the slow-4 or slow-5 frequency bands reflecting gray matter-related neural activity. According to a prior fMRI study on children with CSNHL, this may be related to the functional reorganization of auditory and language brain areas caused by hearing deprivation [47].

The nomogram could be potentially used in clinical practice with various approaches. For instance, the nomogram may help the clinicians to provide a more informed consent to the patients who were going to under CI. From the nomogram, we could identify the patients who may have a good or a poor hearing rehabilitation outcome and this information may be related to the patients and their families prior to CI for them to make an informed decision. On the other hand, the information from the nomogram may also help the multidisciplinary team caring for the children with CSNHL to provide personalized treatment. For the children who have a higher total score from the nomogram indicating a higher probability for a poor hearing rehabilitation, more intense rehabilitation, longer duration of hearing support, broader range of intervention to improve quality of life and closer follow-up are critical to achieve a better outcome for the vulnerable children with this severe debility.

There were several limitations to this study. First, this was a single center study with a modest sample size and validation was performed internally using bootstrap resampling. A multi-center study with external validation is needed to validate the prediction model and make it more generalizable. Second, the patient group and the control group were not matched by age with the control group being significantly older than the children with CSNHL. We found it challenging to recruit younger controls to match the patients with CSNHL because of the difficulties to obtain consents from the parents and legal guardians to have their healthy young children undergo sedation for neuroimaging. Nevertheless, lessons were learned from this study, which should be helpful for our ongoing and future clinical trials studying CSNHL in children. Third, we focused analysis on the neuroimaging data acquired prior to CI and we did not have follow-up rs-fMRI data after CI. It was because the materials of CI and surgical changes cause extensive susceptibility artifacts and render the post-surgical rs-fMRI scans suboptimal for detailed analysis.

## 5 Conclusions

In summary, we have developed a novel nomogram with robust performance for predicting rehabilitation outcome in children with CSNHL after CI. In addition, several independent risk factors, including white matter lesions and modifications in brain function within particular frequency and brain regions, were identified based on the model. The nomogram may be potentially useful as a prognostic tool to support in clinical decision making for personalized treatment if validated in a prospective large-scale multicenter study.

## Funding

This work was partly supported by the 10.13039/100014717National Natural Science Foundation of China (Grant Numbers 82260344); the 10.13039/501100004607Natural Science Foundation of Guangxi, P.R. China (Grant Numbers 2020GXNSFAA259047); and the Clinical Research “Climbing” Program of the First Affiliated Hospital of Guangxi Medical University (Grant Numbers YYZS2020021).

### Ethics approval statement

All subjects participated voluntarily. The participants provide their written informed consent to participate in this study. The Declaration of Helsinki was adequately addressed, and the study was approved by the Medical Ethics Committee of the First Affiliated Hospital of Guangxi Medical University (register number [Ethics]QR2021 No.31).

## Data available statement

The data that support the findings of this study are available from the corresponding author upon reasonable request.

## CRediT authorship contribution statement

**Xi Deng:** Writing – original draft, Visualization, Software, Methodology. **Xueqing Yang:** Writing – original draft, Validation, Investigation. **Meiru Bu:** Investigation, Data curation. **Anzhou Tang:** Supervision, Investigation, Data curation. **Huiting Zhang:** Writing – review & editing, Software. **Liling Long:** Supervision, Resources. **Zisan Zeng:** Supervision, Project administration. **Yifeng Wang:** Writing – review & editing, Software, Methodology, Data curation. **Ping Chen:** Supervision, Project administration, Investigation, Data curation. **Muliang Jiang:** Writing – review & editing, Supervision, Investigation, Funding acquisition, Data curation. **Bihong T. Chen:** Writing – review & editing, Supervision, Methodology.

## Declaration of competing interest

The authors declare that they have no known competing financial interests or personal relationships that could have appeared to influence the work reported in this paper.

## Abbreviations

BCNC =: bony cochlear nerve canal
CAP =: category of auditory perception
CI =: cochlear implantation
CSNHL =: congenital sensorineural hearing loss
HRCT =: high resolution computed tomography
IAC =: internal auditory canal
LFO =: low frequency oscillation
MRI =: magnetic resonance imaging
ROC =: receiver operating characteristic
rs-fMRI =: resting-state functional MRI
WML =: white matter lesion
